# Effect of Esterification Conditions on the Physicochemical Properties of Phosphorylated Potato Starch

**DOI:** 10.3390/polym13152548

**Published:** 2021-07-31

**Authors:** Jacek Rożnowski, Lesław Juszczak, Barbara Szwaja, Izabela Przetaczek-Rożnowska

**Affiliations:** Department of Food Analysis and Evaluation of Food Quality, Faculty of Food Technology, University of Agriculture in Krakow, Balicka 122 Str., 30-149 Krakow, Poland; rrjuszcz@cyf-kr.edu.pl (L.J.); barbara.szwa2000@op.pl (B.S.); izabela.przetaczek-roznowska@urk.edu.pl (I.P.-R.)

**Keywords:** potato starch, phosphorylation, rheology, DSC, retrogradation

## Abstract

The aim of this study was to evaluate the effect of the temperature (15 or 45 °C) and the duration (15–120 min) of the modification process on the selected physicochemical, thermal, and rheological properties of phosphorylated potato starch. The modified starches contained 93.6–98.2 mg P/100 g (dry weight basis, d.w.b.). Phosphorylation caused color changes with a total color difference between the starches below 0.55, but these changes were less than those that were recognizable by the human eye. The thermal analysis showed two opposite processes appearing during the modification: the loosening of the structure (dominant among starches obtained at 15 °C) and the strengthening of the structure (dominant among starches obtained at 45 °C). The higher phosphorylation temperature reduced native starch recovery from 140% to 87–116% and increased the hysteresis loop area from −169 to 1040. All of the pastes made from the modified starches showed a weaker tendency for retrogradation (during 21 days of storage) than native starches. The results of the regression analysis conducted between the properties of the starch pastes obtained at 45 °C indicated that the modification time appeared to be a better indicator of the rate of modification progress than the phosphorus content. The PCA (principal component analysis) results made it possible to distinguish starch phosphates obtained at 15 °C from those obtained at 45 °C and those from natural starch.

## 1. Introduction

Starch represents a carbohydrate reserve in plants and is found in leaves, flowers, fruits, seeds, and roots. A starch granule is a densely packed structure with semi-crystalline structures composed of two major starch polymers: essentially linear amylose and branched amylopectin. The specific properties of starches are dependent on their botanical origin and by the size and surface of the starch granule; the lipid, protein, water, and phosphorus content; and the amylose and amylopectin ratio [1,2]. 

For environmental reasons, potato starch is an important industrial raw material in Poland. Its physicochemical properties (large granule size, high polysaccharide purity with very low protein and fat content, and a high phosphorus content) distinguish it from cereal starches, allowing, among other things, to form pastes (at low temperature) with high transparency and viscosity but that are less thermally stable [1,3,4,5,6].

Starch is a very popular and multifunctional compound in the food industry. It mainly offers thickening, gelling, and dispersed system-stabilizing properties; therefore, it is used to improve textural properties [7] and water retention. However, the use of native starch in food products can pose some difficulties because of some negative changes such as retrogradation, syneresis, low stability, and low resistance to shear stress [2,4,8,9,10]. Therefore, modified starches with improved functional properties have been developed via four types of modifications: chemical, enzymatic, genetical, and physical [11]. 

Among the modifications allowed by law for food products, the modifications that are very interesting are phosphorylation [12,13,14], new esterification methods using octenyl succinic acid anhydride (OSA) [15,16], and the enrichment of native and modified starches with mineral ions to increase the proportion of mineral compounds in the diet [13,15,17].

Starch is chemically phosphorylated with phosphoryl chloride, sodium trimetaphosphate (STMP), sodium tripolyphosphate (STPP), or sodium or potassium orthophosphate [11]. The incorporation of the orthophosphate groups into the starch structure, resulting in the formation mono- or distarch phosphates, has been widely used in the industry to modify its functional properties [1,11] Advances in genetic engineering technology have enabled the genetic modification of starch in planta. Changes in the enzymes of the starch biosynthetic pathway affect the starch structure and the possibility of using products from genetically modified plants in food processing. This transgenic technology reduces or eliminates the use of environmentally hazardous chemical and/or enzymatic modifications [11].

Phosphorus is a constituent element of a potato starch granule. Either by its presence in the covalently bound orthophosphate groups or by binding metal ions, it imparts ion exchange properties to starch [4,8,18]. The granules of native starch are indigestible due to an internal structure of starch molecule occurring in a relatively low moisture. The orthophosphate group in mono- and distarch phosphates are liberated in the metabolic pathways of the human organism [19]. Cross-linked starches exhibit resistance to digestive enzymes and behave physiologically like soluble fibers [20]. 

Owing to their functional properties, the starch phosphates E 1410 (monostarch phosphate), E 1412 (distarch phosphate), E 1413 (phosphated distarch phosphate), E 1414 (acetylated distarch phosphate), and E 1442 (hydroxypropyl distarch phosphate) were approved for use as food additives by the European Union [21] and for use in the USA [22]. According to the EU Commission Regulation 1129/2011 [23], starch phosphates were authorized as additives for food uses at *quantum satis* levels.

However, the use of chemical ingredients in food production is causing concern in a growing number of consumers. This is due to the presence of many allergies of unknown aetiology and a growing awareness of the risks and effects of such products on the human body. An alternative to genetic or many chemical starch modification methods may be the use of phosphorus compounds. It therefore seems advisable to bring phosphorylation back into the focus of scientific attention and to expand knowledge on this subject.

The knowledge of the transformations occurring in starch during phosphorylation and of the properties of the phosphates formed under different modification conditions will allow the selection of modification conditions to optimize the production costs while obtaining the most favourable functional properties of the phosphates.

The use of STMP for the preparation of distarch phosphates allows the modification to be conducted under mild conditions using low-cost reagents that are safe for humans and for the environment.

Given the above, this study aimed to evaluate the effect of the temperature (15 or 45 °C) and the duration (15–120 min) of the modification process on the selected physicochemical, thermal, optical and rheological properties of phosphorylated potato starch.

## 2. Materials and Methods 

### 2.1. Materials

Commercial potato starch with average molecular weight of amylose at 9.0 × 10^6^ and amylopectin with an average molecular weight of 9.4 × 10^7^ was purchased from Potato Industrial Enterprise PEPEES S.A. (Łomża, Poland). 

Salts (analytical grade) were purchased from Avantor Performance Materials Poland S.A. Gliwice Poland (Na_2_CO_3_, NaCl) and Sigma Aldrich (STMP—sodium trimetaphosphate). Urea-dimethylsulphoxide (UDMSO) solution was prepared from dimethylsulphoxide (Avantor, Poland) and urea (Sigma Aldrich, St. Louis, MO, USA).

### 2.2. Modification Procedure

Starch modification began by melting STMP (2.7 g) with 440 mL of deionized water (15 °C or 45 °C) with Na_2_CO_3_ (5 g) and NaCl (5 g). The pH of the solution was adjusted at level 10.5 using 5% solutions of HCl or NaOH. The starch (220 g d.w.b.) was dispersed in the solution, and the pH was then readjusted. Next, the obtained solution was mixed at a controlled temperature (15 °C or 45 °C) and time (15, 30, 45, 60 or 120 min). The crude product was filtered through a 1G Schott funnel, was washed with water, and was air-dried. The dried starch was ground using a mortar grinder RM 200 (Retsch, Germany) and was passed through a 200 µm sieve using a vibratory sieve shaker AS 200 (Retsch, Germany) before the analysis.

**Figure 1:**
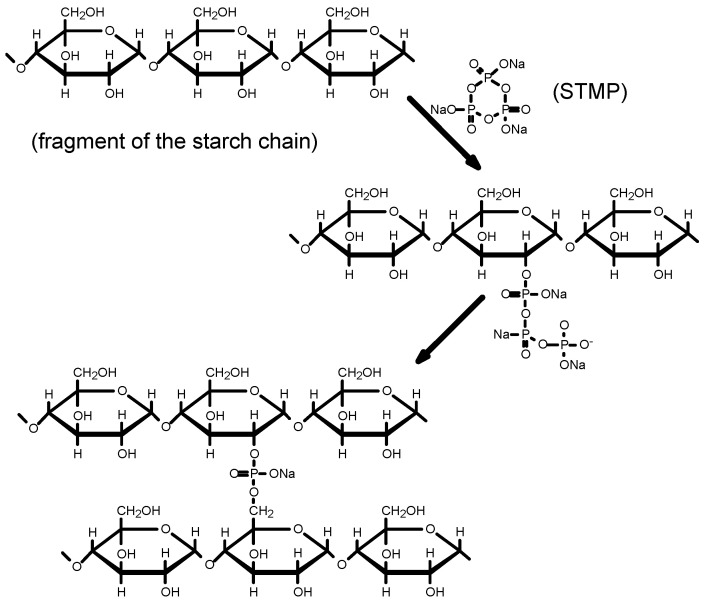
Mechanism of starch esterification caused by STMP in alkaline (pH = 10.5) suspension.

According to Lim and Seib [24], the starch phosphorylation mechanism using STMP begins with the ring-opening of the STMP during the reaction with the C2, C3, or C6 hydroxyl group of the anhydroglucose units of the starch chain (Figure 1). In the next stage, the resulting starch tripolyphosphate reacts at pH = 10.5 with anhydroglucose units from other starch chains to produce distarch phosphate and pyrophosphate.

During modification, cross-linking bonds may be formed between C2, C3, and C6 atoms [25].

### 2.3. General Methods

The moisture content was calculated as the percentage weight loss of a 1 g sample heated at 130 °C for 1 h and cooled to room temperature in a desiccator [26]. 

The total phosphorus content in native and phosphorylated starch dry matter was determined after mineralization in a hot mixture of HNO_3_ and H_2_SO_4_ using a Wet Digester B-426 (Büchi, Switzerland). A reaction with (NH_4_)_6_Mo_7_O_24_ and ascorbic acid was followed by the spectroscopic measurement of the intensity of the blue color at a wavelength of 825 nm (V-530 Jasco, Tokyo, Japan) [27]. 

The amylose content in the starch dry matter was determined using a V-530 spectrophotometer (Jasco, Japan), according to the methods of Morrison and Laignelet [28]. The apparent amylose content was calculated from the blue value (defined as the absorbance of 10 mg of anhydrous starch in 100 mL of diluted I_2_-KI solution at 635 nm and 20 °C). 

The total fat content was determined using petroleum ether (b.p. 40–60 °C) and was determined according to the methods of Soxhlet [29], whereas the total protein content (N × 6.25) was determined using the Kjeldahl method [30] in order to assure a low lipid (<0.3%) and protein (<0.5%) content. The detailed data are not shown in this report.

### 2.4. Color Analysis

The color of the starches under investigation was determined using a Color i5 spectrophotometer (X-Rite, Grand Rapids, MI, USA). D65 illuminant was applied for the d/8 geometry. The color was determined in the specular excluded mode (SPEX) for an additional observer (CIE 1964). The CIE L*, a*, b*, C*, and h° coordinates [31] were determined using the X-Rite Color Master software.

The CIE 1976 total color difference (ΔE) between two color stimuli was calculated as the Euclidean distance between the points representing them in the CIE L*a*b* color space [31]:

ΔE = [(ΔL*)^2^ + (Δa*)^2^ + (Δb*)^2^] ^1/2^ (1)

Based on the experimentally determined L*a*b* coordinates, the whiteness indexes were determined using the formulas developed by CIE (WI_C_), Uchida (WI_U_), and Judd (WI_J_) [32,33]:

WI_C_ = Y + 800 · (0.3138 − x) + 1700 · (0.3310 − y) (2)

WI_U_ = WI_C_ − 2 · (TI)^2^ (3)

WI_J_ = 100 − [(100 − L*)^2^ + (a*)^2^ + (b*)^2^]^1/2^ (4)

Finally, the yellowness index (YI) and the tint index (TI) were determined:

YI = 100 · (1.3013 · X − 1.1498 · Z)/Y(5)

TI = 900 · (0.3138 − x) − 650 · (0.3310 − y)(6)

where: X, Y, Z, x, y are color coordinates in the CIE Yxy color space [31,32].

### 2.5. Differential Scanning Calorimetry (DSC)

Thermal properties of the modified starches were analyzed using a Differential Scanning Calorimeter Phoenix 204 F1 (Netsch, Selb, Germany) that was calibrated using iridium. An empty pan was used as a reference.

#### 2.5.1. Gelatinization

Starch (3.5 mg) was loaded into an aluminum pan (Netzsch, Germany) and distilled water was added using a micropipette to create a suspension with a 75% moisture content. Samples were hermetically sealed and equilibrated for 24 h at room temperature before being heated in the DSC. The measurements were conducted at a heating rate of 10 °C/min from 20–100 °C. The enthalpy of the phase transitions (ΔH_1_ J per g d.w.b. of starch) was measured from the endotherm of DSC thermograms using the Netzsch Proteus–Thermal Analysis software (Netzsch, Germany) based on the mass of the dry solids. The onset temperature (T_o1_), peak temperature (T_p1_), and conclusion temperature (T_c1_) were calculated. The gelatinization temperature range (ΔT_1_) was calculated as T_c1_-T_o1_. The peak height index (PHI_1_) was computed from the ΔH_1_/(T_p1_-T_o1_) ratio as described by Kaur [3]. The reported values are the means of four measurements. 

#### 2.5.2. Retrogradation

After being heated for gelatinization analysis, the samples (in the original sealed pan) were cooled to 4 °C and were stored at this temperature. After a week, the stored samples were heated from 20–100 °C at 10 °C/min (the second run). The enthalpy (ΔH_2_), onset temperature (To_2_), peak temperature (Tp_2_), and conclusion temperature (Tc_2_) were determined. The transition temperature range of the melting of recrystallized amylopectin (ΔT_2_), the peak height index (PHI_2_), and the retrogradation ratio ΔH_2_/ΔH_1_ were calculated. The reported values are the means of four measurements.

### 2.6. Rheological Measurements

Rheological analysis was performed using a rotational rheometer Rheolab MC1 (Physica Masstechnik GmbH, Ostfildern, Germany) with coaxial cylinders (gap 2.12 mm) as a measuring system programmed using the US 2000 software. The rheological properties were determined for 5% (*w/w*) starch pastes prepared in water (at 95 °C for 30 min with continuous stirring). The produced pastes were immediately placed into the rheometer’s measuring system and were thermostated at 50 °C prior to determination.

#### 2.6.1. Flow Curves

The flow curves were obtained at 50 °C according to the program, with the shear rate increasing from 1 to 300 s^−1^, at constant shear rate 300 s^−1^, and with the shear rate decreasing from 300 to 1 s^−1^.

The curves were fitted using the Ostwald de Waele model, the Herschel–Bulkley model, and the Casson model. The publication discusses the parameters of the Herschel–Bulkey model because it was characterized as the best fit to the measurement data (R^2^ ≥ 0.997):

(7) $$\tau =\tau_{0}+K\cdot {\dot{\gamma }}^{n}$$

where τ is the shear stress (Pa), τ_0_ is the yield stress (Pa), $\dot{\gamma }$ is the shear rate (s^−1^), K is the consistency coefficient (Pa·s^n^), and n is the flow behavior index.

Table S2 in the Supplementary Materials provides the coefficients of the fitted equations of the Ostwald de Waele model (R^2^ ≥ 0.994) and the Casson model (R^2^ ≥ 0.985), which taken for correlation analysis. 

#### 2.6.2. In-Shear Structural Recovery Measurement

The in-shear structural recovery of the paste samples was determined according to a procedure modified from Mezger [34]. The test was performed at 50 °C, following a three-step program with a constant shear rate of 1 s^−1^, a constant shear rate of 300 s^−1^, and a constant shear rate 1 s^−1^ in the subsequent steps.

The recovery (R) was calculated as the percentage of the mean apparent viscosity obtained during the last 450 s of the third step (η_ap3_) based on the value determined in the first step (η_ap1_):

R = η_ap3_ · 100%/η_ap1_ (8)

### 2.7. Turbidimetric Analysis of Susceptibility to Retrogradation

The turbidity of the 2% (*w/w*) pastes was measured using the Jacobson et al. [10] using our own method of modification [14]. Starch dispersions prepared in deionized water using a mechanical overhead stirrer. They were stirred at 25 °C for 10 min and were then kept in a water bath at 95 °C for 30 min. The temperature was then lowered to 25 °C for 20 min under continuous stirring. 

The obtained batch of paste was poured into eight 2 mL plastic cuvettes (1 mm thick). The initial turbidity (T_0_) of the starch pastes was determined at 640 nm against a distilled water blank using a V-530 spectrophotometer (Jasco, Japan). The remaining seven samples were stored at 8 °C for three weeks. After 1, 3, 5, 7, 10, 14, and 21 days of storage, the cuvettes with the samples were equilibrated for 30 min at 25 °C, and the turbidity was measured.

The normalized turbidity (NT_x_) after x days of storage was calculated as:

NT_x_ = (T_x_ − T_0_) · 100%/(T_21_ − T_0_) (9)

where T_x_—turbidity after x days of storage, T_0_—turbidity before storage (0 day), and T_21_—turbidity after 21 days of storage.

### 2.8. Statistical Analysis

The analyses were performed in three or four parallel repetitions. Tables present mean values and standard deviations. A one-way analysis of variance with a significance level of 5% was conducted, and Tukey’s HSD (honestly significant difference) test was applied to determine the differences between means using the statistical package Statistica 11 (StatSoft Inc., Tulsa, OK, USA). 

Pearson’s correlation coefficients were determined to evaluate the relations between the modification time, phosphorus content, and the rheological and thermal variables in the three distarch phosphates groups, i.e., those obtained at a lower temperature (L), those obtained at a higher temperature (H), and all phosphates (L + H).

A principal component analysis (PCA) was used to provide a ready means for visualizing the similarities or differences among the analyzed starches.

## 3. Results and Discussion

### 3.1. General Methods

The physical properties of starch (structure, water solubility, retrogradation, viscosity) are vital indicators of starch quality and applicability. These properties are related to the non-carbohydrate components in starch: water, phosphorus, amylose, protein, and fat. The physicochemical properties of native potato starch and derived modified starches are shown in Table 1. Water content is one of the main factors determining the chemical, biochemical, and physical processes in food. The modification reaction was conducted in a water suspension at either a lower temperature (15 °C—L-starches) or a higher temperature (45 °C—H-starches). After the modification, all distarch phosphates were air dried until reaching the equilibrium moisture content. The water content of the analyzed starches ranged from 13.2% to 15.1% (Table 1) and was similar to the mean value of 17.5% reported for potato starch by Przetaczek et al. [35] and the value of 19% reported by Swinkels [8]. The national legislation and EU regulations [21] define purity criteria and have stipulated that the water loss after drying should be no more than 21.0% for modified potato starches. All of the tested starches met this requirement. 

The phosphorylation of potato starch was performed using reactions described by Lim and Seib [24]. According to them, distarch phosphates are obtained in basic media. In distarch phosphates, orthophosphate groups serve as the bridges between two chains (intermolecular bridges) or between two hydroxyl groups of the same chain (intramolecular bridges).

Native starch contained 85.2 mg P/100 g and 23.5% amylose (Table 1), which is typical of potato starch [5,8,9,35,36].

When the modifying phosphorus was incorporated into the starch, the total phosphorus content increased with the process temperature to 93.6–97.1 mg P/100 g in the L-starches and to 95.9–98.2 mg P/100 g in the H-starches. Because the results of an earlier work by Fortuna [37] indicated that the hydrolysis of covalent phosphate monoesters accompanied the modification process in an alkaline suspension, it was expected that the number of cross-linkages would be greater than is apparent from the increase in phosphorus content. At the same time—as reported by Yoneya et al. [38]—the linking process need not be linearly dependent on only one modifying agent. 

In accordance with the EU legislation [21] and the FAO/WHO [39] guidelines on the specification and purity criteria of modified starches, the permissible phosphate residue in a distarch phosphate (E1412) is ≤ 0.5% (as phosphorus) for wheat or potato starch. Based on those regulations, it can be concluded that the formulations of the modified starches obtained in this study comply (in terms of phosphorus content) with the EU and FAO/WHO requirements and hence can be used in food technology.

The apparent amylose content in the starches was very similar (23%), but three starches (H45, H60, H120) were insoluble in UDMSO, and their analysis could not be continued. The poor solubility of some starches in UDMSO may have been due to the greater degree of cross-linking. According to Singh et al. [4], Pycia et al. [6], Zhu and Cui [40], and Sulaiman et al. [41], the amylose content in potato starch granules ranged from 19.1 to 31.0%, which is caused by crop fertilization with phosphorus compounds, a large variety of cultivars, and various methods used to determine amylose content. 

The fat and protein content were determined to assess the purity of the starch raw material (N-starch). The total fat content of the starch did not exceed 0.3% (d.w.b.), and the protein content did not exceed − 0.5% (d.w.b.; N × 6.25).

### 3.2. Color Analysis

The D65 illuminant used (average daylight including UV, T = 6504 K) is the preferred illuminant for color description [31]. All of the tested starches were characterized by very high brightness, with L* values exceeding 92.5 (Table 2). Their modification resulted in a slight decrease (from 93.41 to 92.66–93.12) in their brightness. The chromaticity coordinates of the samples were in the second quadrant of the a*b* plane with hue angle (h°) values between 92.28 and 96.18. Potato starch containing very small amounts of fatty and protein compounds was characterized by low chroma (C* < 2.32). A slight negative value of the green component (−0.23 < a* < −0.09) in combination with the values of the b* component (2.05 < b* < 2.32), which were 10–40 times higher, resulted in a decisive influence of the yellow component (b*) on the hue and the saturation of the color. The conducted research showed no significant influence of temperature and modification time on the values of the L*, a*, b*, C*, and h° parameters. The statistical analysis results, including the standard deviation, are presented in Table S1 (Supplementary Materials).

Whiteness is a sensorially important property of color appearance in various industries. White is the attribute of a visual sensation according to which a given stimulus appears to be void of any hue or grayness. Its quantitative analytical measure is a whiteness index, the higher value of which indicates the greater whiteness of the sample. The value of the whiteness index (WI) of native starch determined according to the equation developed by CIE was 73.70. Phosphates obtained at the lower temperature showed a 1–3% lower WI, while the WI of those obtained at the higher temperature was similar to native starch. The results of the whiteness analysis using the equation developed by Uchida were lower by 1.5 units. In the case of the Judd’s equation, the native starch was the most white (WI_J_ = 92.98), while all of the modified starches were darker (WI_J_ = 92.37–92.82).

A yellowness index parameter (YI) is an indicator of the yellowness contributing to the visual evaluation of starch products [32] and needs to be considered when considering consumer acceptance. When starch is used as a food additive, it is expected to have a low YI value in order to preserve the organoleptic acceptability of a food product’s color. The analyzed samples (Table 2) were characterized by low yellowness (3.82 < YI < 4.36), which was not significantly affected by the modifications. Therefore, the obtained modified starches should not change the customer’s sensory impression when they are consuming products using starch as a thickening substance. The determined negative values of the hue index (−0.72 < TI < −0.42) as well as the negative values of the b* component indicated that the starches had a slightly green hue.

Euclidean distances in the CIE L*a*b* color space can be used to approximately represent the perceived magnitude of the color differences between object color stimuli of approximately the same size viewed in identical white to middle-grey surroundings. The total color differences (ΔE) between the starches presented in Table 3 do not exceed 0.42, which indicates that the determined differences are imperceptible to the human eye, even by an experienced analyst.

### 3.3. DSC Analysis of Starch

When starch is heated in an excess of water, its gelatinization occurs with a heat flow change. The results of the DSC analysis of the starch gelatinization test are summarized in Table 4. The phase transition of the starch from the ordered structure (granular) to the random state (coil) was represented by endothermic peaks between 59.9 and 72.9 °C [6,36,40,41,42]. The DSC profiles of L-starches were very similar; T_o1_, T_p1_, and T_c1_ did not depend on the modification time. Increasing the phosphorylation temperature increased T_o1_ and T_p1_ by at least 0.5 °C (compared to the L-starches). Moreover, those temperatures increased along with the modification time. The onset temperature ranged from 61.05 °C (H15) to 62.8 °C (H120), whereas the peak temperature reached 66.0 and 67.3 °C (H15 and H120, respectively). 

During modification, starch is mixed in an aqueous medium, which may cause the partial disordering of its structure (combined with the hydrolysis of some phosphate groups), which will be counteracted by the cross-linking effect. Since the melting point of starch crystallites is influenced by the surrounding amorphous regions, an earlier start of gelatinization (T_o1_) in the L-starches (compared to the N-starch) indicates the loosening of the structure during phosphorylation. In the case of H-starches, the gradually increased cross-linking (along with the extension of the modification time) ensured a protective effect. As the gelatinization temperatures (such as T_o1_ and T_p1_) reflect the structure ordering degree in the starch granules, it may be assumed that the H-starches were harder. As suggested by Gunaratne and Hoover [43], the difference in the ΔT values of the starches indicates the various heterogeneity of the crystallites within L- and H-starches.

The modification conditions did not affect the conclusion of gelatinization; therefore, the gelatinization range decreased. The gelatinization enthalpy of the native starch (21.86 J/g d.w.b.) and the modified starches (21.37–23.83 J/g d.w.b.), and the peak height index (4.77 and 4.13–5.15, respectively) did not differ.

After the gelatinization analysis, the samples were stored for 7 days, and the DSC analysis was performed again. The results of the DSC analysis of the retrogradated samples are summarized in Table 5.

All of the modified starches showed wide and low endothermic peaks between 38 and 72 °C. Similar results were reported by Singh et al. [4], Morikawa and Nishinari [44], Liu et al. [45], and Przetaczek-Rożnowska and Fortuna [12] for native potato starch. The onset temperature ranged from 38.6 to 46.5 °C and increased over the modification time. The modification temperature had no effect on T_o2_. A higher modification temperature increased the peak temperature by about 1–2 °C. The thermal transition of all of the samples ended at similar temperatures (70.3–71.9 °C). Extending the phosphorylation time reduced the width of the DSC peaks (ΔT_2_), while the modification temperature had no effect. The enthalpy of re-gelatinization was similar to data reported for native starch by Singh et al. [4] and Pycia et al. [6] and was a little higher than data reported for native potato starch [40,41,46] despite the decreased starch content [47]. 

### 3.4. Flow Curves

A starch paste is a diphase system of amylose (a continuous phase colloidal amylose solution) and starch granules composed mainly of amylopectin (a dispersed phase). Rheological properties of such systems depend on many factors, including the type of starch, the starch content, the paste preparation method, and the measurement temperature [44,48,49].

**Figure 2:**
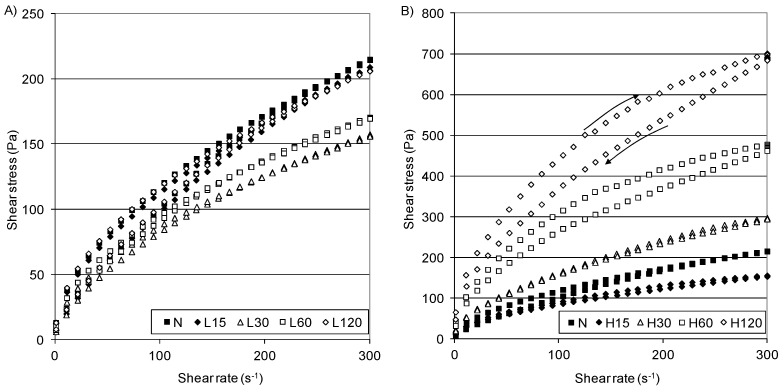
Flow curves of native starch and distarch phosphate pastes: (**A**) L-starches, (**B**) H-starches.

The rheological analysis of the starch pastes can be investigated using several methods [33]. The most common is providing flow curves arranged in the form of a loop, the area of which is considered as a measure of the energy delivered on shearing [50,51]. Flow curves plotted for native and modified starches and the hysteresis loop area values are shown in Figure 2, while the parameters of the rheological Herschel–Bulkley model are given in Table 6. All of the pastes exhibited a non-Newtonian, shear-thinning flow with a tendency to yield stress, i.e., their behavior was typical of systems of this type [40,51]. Figure 2 A shows the flow curves of the native and L-starches. The samples that were modified at the low temperature revealed slightly lower (158–206 Pa) shear stress values (at 300 s^−1^) than the native starch (215 Pa). This is confirmed by calues that are similar (only with small differences) to those of the calculated rheological models (Table 6). Starch pastes obtained at the higher temperature exhibited higher shear stress values than those produced at lower temperatures, and these values increased from 155–700 Pa at 300 s^−1^ with the modification time (Figure 2B).

As was observed, a curve with a decreasing shear rate at the lower shear rate was above the curves obtained for the increasing shear rate (anticlockwise). Therefore, the thixotropy hysteresis area (Table 6) was negative (antithixotropy), while a small thixotropy was observed at the higher shear rate in some of them [51].

All starch pastes tended to the yield stress: 5.36 Pa (N-starch), 2.25–4.61 Pa (L-starches), and 2.51–20.88 Pa (H-starches). For the starch pastes obtained at 45 °C, the consistency coefficient (K) increased 3–11 times compared to the starches obtained at 15 °C (Table 6), and this increased with the modification time, ultimately reaching 43.29 Pa·s^n^ (H120). The higher τ_0_ and K of the H-starches compared to the N-starch may be attributed to the longer chain length of the cross-linked starches as observed in the DSC profiles. The flow index of all of pastes was lower than one (n < 1), pointing to their shear-thinning character, but there was no regularity in the course of changes in n against the modification time, although H-starches had significantly lower n than the N-starch and L-starches.

### 3.5. In-Shear Structural Recovery Measurement

**Figure 3:**
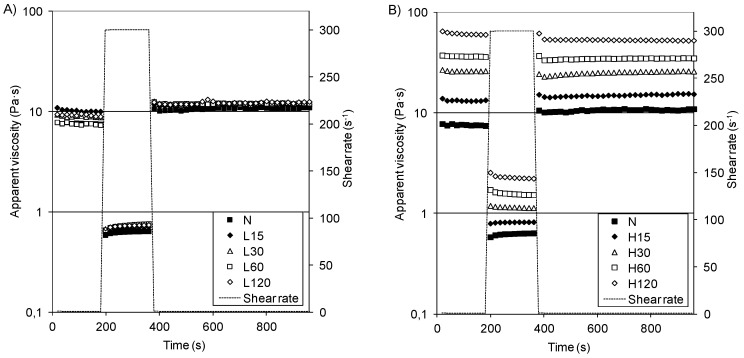
In-shear structural recovery curves of pastes of native starch and distarch phosphates: (**A**) L-starches, (**B**) H-starches.

In-shear structural recovery measurements were performed to evaluate the possibility of paste recovery after the destruction of their original structure due to a sudden change in the shear rate [5,34,50]. Changes in the apparent viscosity were presented in Figure 3A,B. During measurements with a low shear rate (1 s^−1^), the starch pastes obtained at the temperature of 15 °C showed a low apparent viscosity (7–11 Pa·s) that was similar to that of native starch paste (1 Pa·s), while the apparent viscosity of starch pastes obtained at a higher temperature increased noticeably over the modification period (14–63 Pa·s).

After the shear rate increase or decrease, all of the analyzed pastes quickly reached a stable viscosity. The recovery percentage of their structures is shown in Table 6. Starches N and L showed a recovery of 113–159%, which indicates a different starch paste structure. The H-starches were more resistant to the mechanical influences; however, their recovery diminished with an increasing phosphorus content (R = −0.9299) and with the modification times (R = −0.7813).

### 3.6. Turbidimetric Analysis of Susceptibility to Retrogradation

Light transmittance is one of the essential starch paste indicators. The process of retrogradation is associated with the release of the insoluble starch form from the suspension, leading to a reduction in the light transmission of the paste through the progress of retrogradation [2,10].

**Figure 4:**
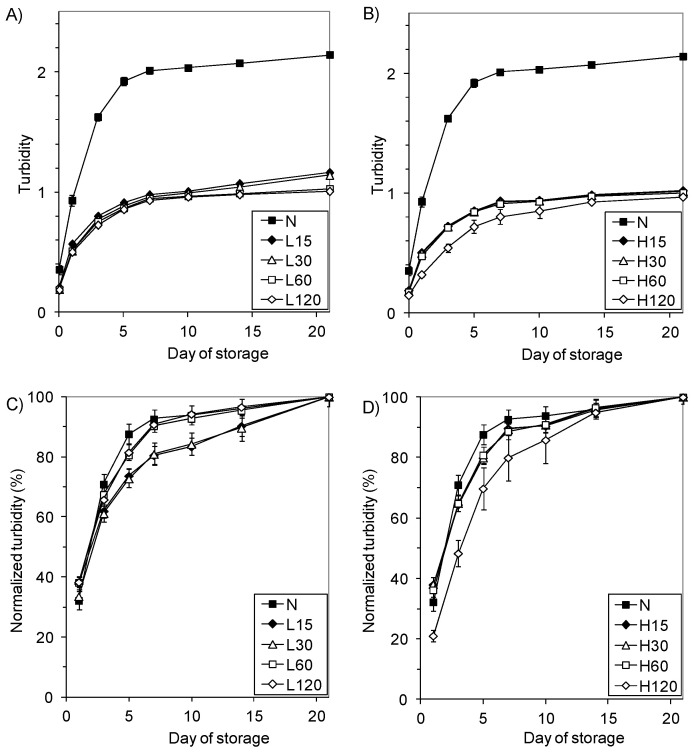
Time dependence of turbidity of 2% pastes stored at 8 °C: (**A**) turbidity of L-starches, (**B**) turbidity of H-starches, (**C**) normalized turbidity of L-starches, (**D**) normalized turbidity of H-starches.

Changes in the turbidity of the starch pastes during storage at 8 °C (Figure 4A,B) are depicted in the curves increasing with a trend to reach the plateau.

The turbidity of the N-starch was lower than the values calculated by Morikawa and Nishinari [44] and Craig et al. [52], which may be associated with the different sodium ion content in starch. The monoester phosphate groups present in potato starch repel each other, thus hindering association (related to the formation of intra- and intermolecular bonds) and increasing the clarity of the starch glues. In the presence of sodium cations, the repulsive effect of the negative phosphate groups is limited. This effect is much weaker in the case of other starches [52].

The pastes made with modified starches (even starches insoluble in UDMSO) are twice as transparent as the native starch paste. The transparency increased with phosphorylation process duration, and the starches that were modified at a higher temperature (H-starches) had a lower turbidity than the corresponding L-starches. On the other hand, literature data [52] show that during the stronger cross-linking of starch caused by phosphorus oxychloride, the clarity of the pastes decreases. Thus, the optical properties of starch result from two opposing factors: the removal of sodium ions from starch associated with the partial hydrolysis of the monostarch phosphate during modification, and cross-linking, which impedes particle dissociating. In the case of a low degree of cross-linking, the decisive factor seems to be the influence of the cations than with a higher-degree cross-linking effect. It can be expected that the granules of the cross-linked starches will not break down entirely, and their residues (so-called “starch ghosts”) will float in the paste, increasing its turbidity in the same way throughout the storage time. Nevertheless, the turbidity of the modified starch pastes was tangibly lower than that of the native starch pastes.

To compare the rate of the process of opacification during storage, the curves of relative turbidity progressed as the normalized turbidity curves were plotted (Figure 4C,D). They show a rapid increase in the opacity of native starch compared to the modified starches, and slight differences in the progress only occurred at the beginning of storage. The sharp increase in turbidity in the first day of storage reflects the formation of networks that are mainly due to the interactions between the amylose chains (leached out during gelatinization) and show that the retrogradation of amylopectin is a slow and ongoing process [2]. Since the native and modified starches did not differ in amylose content, the slower increase in turbidity in the initial storage time of the modified starch pastes should be attributed to the electrostatic repulsion of the elongated cross-linked amylose chains containing polar orthophosphate groups, which hinder the formation of the network.

### 3.7. Correlation Analysis and PCA

The statistically significant coefficients of Pearson’s correlation among the physicochemical parameters and the phosphorus content and the modification time of various starches (L or H) and all of modified starches (L and H) are shown in Table 7. 

In the case of starches obtained at the lower temperature, no significant correlation was found between the parameters of the rheological models and the modification time or the phosphorus content. This may be due to mild modification conditions that do not cause major changes in the starch granule (e.g., in the phosphorus content). Strong correlation relationships occurred in starches obtained at a higher temperature (H-starches), and higher values of the correlation coefficients for the physicochemical parameters with the modification time than with the phosphorus content indicate that the modification time has a greater impact on the properties of starch and its pastes. The coefficients of correlation determined for all of the modified starches (L + H) showed an opposite tendency, which indicates a multitude of processes taking place during the modification and prompts the development of models based on many variables. In turn, the weaker correlations found for all of the modified starches (L + H) than for the H-starches emphasize the influence of the modification temperature. The discussed rheological parameters were positively correlated, except for the flow indices (−0.68, −0.79, −0.89). The thermal properties variables (T_o_, T_p_) obtained after the gelatinization and retrogradation analysis were highly positively correlated with the modification time and the phosphorus content and were negatively correlated with the temperature range, while the conclusion temperature was not strongly correlated with them. No significant correlation between the discussed parameters and the measured enthalpy may be due to the two opposite effects described by other authors.

According to Bidzińska et al. [53], the enthalpy decreases for reference samples (blind samples obtained without a phosphorus-containing reagent) but increases for phosphates, which seems to imply that phosphorylation is accompanied by some cross-linking of the starch chains. An opposite effect was described by Wootton and Bamunuarachchi [54], according to whom the decrease in gelatinization enthalpy of the metal-fortified starch was mainly caused by the incorporation of new substituents into an interior structure of the granules. The peak height index was positively correlated to the discussed parameters. 

**Figure 5:**
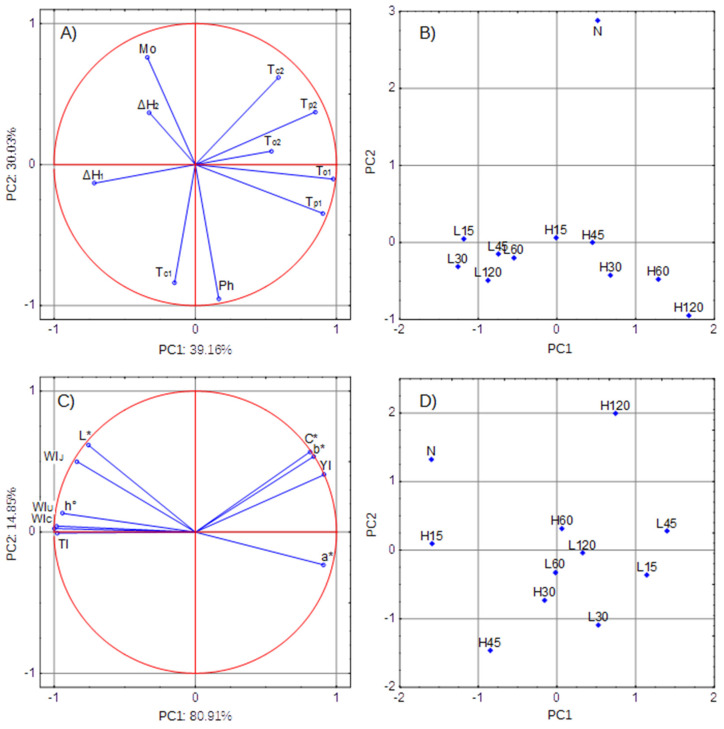
Principal component analysis: PCA: (**A**) distribution of parameters of the internal structure of the granule-loading plot (Mo-moisture, Ph-phosphorus content), (**B**) distribution of starch samples based on the internal structure-score plot, (**C**) distribution of color parameters-loading plot, (**D**) distribution of starch samples based on color-score plot.

The PCA allows the reduction of the number of variables using the correlation between the parameters describing the properties of the object. Reducing the number of variables makes it easier to visualize differences between the compared samples. Figure 5A shows the distribution of parameters describing the internal structure of the granule (parameters of thermodynamic transformations as well as water and phosphorus content). The first two principal components (with Eigenvalues of 3.91 and 3.03) explained 69.19% of the total data variance. Because the current analysis is based on correlations, the largest possible PCA coordinate is equal to 1.0. Figure 5A shows that the thermodynamic pasting parameters (ΔH_1_, T_o1_, T_p1_) were related to the first component, and that the composition parameters (Mo and Ph) contributed significantly to Component 2. The parameters describing the retrogradation process significantly contributed to the values of both principal components. Figure 5B shows the distribution of starch samples on the plot of Components 1 and 2. It shows that the starch modification temperature was the factor that grouped the samples into 3 populations: L-starches, H-starches, and N-starch.

The second part of the principal component analysis involved the color analysis of the outer layers of the starch granule. Figure 5C shows the distribution of color parameters: both components explained as much as 95.75% of the total variance. The figure shows that C*, b*, and YI were strongly positively correlated with each other and significantly contributed to the formation of both components. Similar observations were made for the parameters L* and WI_J_. In turn, strongly positively correlated WI_U_ and WI_C_ as well as h° and TI contributed to the formation of Principal Component 1. As shown in Figure 5D, there were differences between the L-starches, H-starches, and the N-starch, but the division into groups was not that clear.

## 4. Conclusions

The study showed that the duration of phosphorylation affected most of the rheological and thermal properties of starch. It appeared to be a better indicator of the rate of modification progress than the phosphorus content. 

The phosphorus content and the moisture content of the obtained starch phosphates met the EU legal requirements in the field of substances added to food and determined that the phosphorylation imparted valuable technological properties to the starch.

The modification process was accompanied by the loosening of the starch granule structure (observed in DSC measurements) caused by the reaction medium. All phosphorylated starch pastes showed non-Newtonian flow, shear-thinning, and thixotropic behavior. The hysteresis loop area of paste curves of the samples obtained at 45 °C was much higher compared to the samples of native starch or starch modified at 15 °C. Pastes obtained from phosphorylated starches had a significantly lower recovery values than those obtained from native starch, while the higher temperatures during modification resulted in lower recovery values. The transparency of the pastes increased with phosphorylation process duration, and the starches modified at the higher temperature had a lower turbidity than the corresponding samples modified at lower temperature. Phosphorylation caused color changes, but these changes were imperceptible to the human eye.

The PCA results made it possible to distinguish starch phosphates obtained at 15 °C from those obtained at 45 °C and from native starch. However, further studies are needed to determine the importance of various modification factors on the distribution of phosphate crosslinking bonds as well as the position of the formed crosslinking bonds.

## Figures and Tables

**Table 1:** Chemical composition of starches.

	Moisture (%)	Total Phosphorus Content (mg P/100 g)	Apparent Amylose Content (%)
N *	15.1 ± 0.0 ^a^	85.18 ± 0.59 ^a^	23.53 ± 0.10 ^ab^
L15	13.9 ± 0.0 ^b^	93.59 ± 0.18 ^b^	23.30 ± 0.18 ^a^
L30	14.2 ± 0.1 ^b^	95.25 ± 0.31 ^c^	22.96 ± 0.42 ^a^
L45	14.0 ± 0.0 ^b^	95.22 ± 0.10 ^c^	23.11 ± 0.22 ^a^
L60	13.8 ± 0.0 ^b^	95.49 ± 0.10 ^c^	23.32 ± 0.29 ^a^
L120	14.2 ± 0.1 ^b^	97.07 ± 0.10 ^d^	23.39 ± 0.10 ^a^
H15	13.3 ± 0.2 ^c^	95.92 ± 0.44 ^e^	23.45 ± 0.10 ^b^
H30	13.3 ± 0.3 ^c^	97.29 ± 0.37 ^f^	22.64 ± 0.18 ^c^
H45	13.2 ± 0.1 ^c^	97.80 ± 0.42 ^g^	**
H60	13.5 ± 0.2 ^c^	98.00 ± 0.14 ^g^	**
H120	13.5 ± 0.1 ^c^	98.24 ± 0.30 ^g^	**

Values followed by the same letters in the same column are not significantly different at α < 0.05. * Code markings of samples are presented in Abbreviations. ** The determination was not possible due to the incomplete dissolution of the sample in UDMSO.

**Table 2:** Color analysis of native starch and distarch phosphates (SPEX, D65, CIE 1964).

	L*	a*	b*	C*	h°	WI_C_	YI	TI	WI_J_	WI_U_
N	93.41 ^a^	−0.23 ^a^	2.11 ^ab^	2.12 ^ab^	96.18 ^a^	73.70 ^a^	3.92 ^ab^	−0.42 ^a^	92.98 ^a^	73.35 ^a^
L15	92.70 ^d^	−0.10 ^d^	2.23 ^cde^	2.23 ^cd^	92.55 ^de^	71.98 ^d^	4.27 ^de^	−0.68 ^ef^	92.37 ^c^	71.04 ^de^
L30	92.66 ^d^	−0.14 ^bcd^	2.16 ^ac^	2.16 ^ac^	93.59 ^cde^	72.18 ^cd^	4.11 ^cd^	−0.60 ^cde^	92.35 ^c^	71.47 ^cde^
L45	92.75 ^d^	−0.09 ^d^	2.27 ^de^	2.27 ^de^	92.28 ^e^	71.89 ^d^	4.36 ^e^	−0.72 ^ef^	92.40 ^c^	70.86 ^e^
L60	92.92 ^bcd^	−0.12 ^cd^	2.13 ^ab^	2.13 ^ab^	93.09 ^de^	72.96 ^abc^	4.05 ^bc^	−0.62 ^def^	92.61 ^bc^	72.20 ^bc^
L120	92.87 ^bcd^	−0.12 ^cd^	2.17 ^ac^	2.18 ^ac^	93.16 ^cde^	72.63 ^bcd^	4.14 ^cd^	−0.63 ^def^	92.55 ^bc^	71.84 ^bcd^
H15	93.12 ^bc^	−0.20 ^ab^	2.04 ^a^	2.05 ^b^	95.57 ^ab^	73.80 ^a^	3.82 ^a^	−0.44 ^ab^	92.82 ^b^	73.41 ^a^
H30	92.80 ^cd^	−0.16 ^abcd^	2.12 ^ab^	2.13 ^ab^	94.32 ^abcd^	72.68 ^bcd^	4.02 ^bc^	−0.54 ^bcd^	92.49 ^c^	72.09 ^bc^
H45	92.81 ^cd^	−0.18 ^abc^	2.04 ^a^	2.04 ^b^	95.05 ^abc^	73.11 ^ab^	3.84 ^a^	−0.48 ^abc^	92.53 ^bc^	72.65 ^ab^
H60	92.90 ^bcd^	−0.15 ^bcd^	2.18 ^acd^	2.19 ^acd^	93.92 ^bcde^	72.67 ^bcd^	4.13 ^cd^	−0.58 ^cde^	92.57 ^bc^	71.99 ^bc^
H120	92.96 ^bcd^	−0.15 ^bcd^	2.32 ^e^	2.32 ^e^	93.79 ^bcde^	72.17 ^cd^	4.39 ^e^	−0.63 ^def^	92.59 ^bc^	71.38 ^cde^

Values followed by the same letters in the same column are not significantly different at α < 0.05.

**Table 3:** The total color difference between starches (ΔE).

	N	L15	L30	L45	L60	L120	H15	H30	H45	H60	H120
**H120**	0.328	0.113	0.339	0.224	0.197	0.171	0.318	0.253	0.317	0.148	0
**H60**	0.318	0.121	0.245	0.188	0.066	0.043	0.258	0.121	0.171	0	
**H45**	0.400	0.279	0.201	0.257	0.154	0.160	0.304	0.090	0		
**H30**	0.413	0.239	0.143	0.169	0.134	0.100	0.332	0			
**H15**	0.113	0.238	0.476	0.445	0.226	0.287	0				
**L120**	0.354	0.142	0.214	0.160	0.068	0					
**L60**	0.303	0.129	0.266	0.226	0						
**L45**	0.501	0.257	0.150	0							
**L30**	0.554	0.352	0								
**L15**	0.266	0									
**N**	0										

**Table 4:** Thermodynamic characteristics of gelatinization of native starch and distarch phosphates.

	T_o1_ (°C)	T_p1_ (°C)	T_c1_ (°C)	ΔT_1_ (°C)	ΔH_1_ (J/g d.w.b.)	PHI_1_
N	61.21 ± 0.13 ^c^	65.80 ± 0.1 ^bc^	72.02 ± 0.25 ^b^	10.79 ± 0.35 ^c^	21.86 ± 0.26 ^ab^	4.77 ± 0.11 ^ab^
L15	59.93 ± 0.17 ^a^	65.58 ± 0.13 ^ab^	72.73 ± 0.24 ^ab^	12.80 ± 0.37 ^a^	23.35 ± 1.60 ^ab^	4.13 ± 0.28 ^a^
L30	60.08 ± 0.17 ^ab^	65.40 ± 0.16 ^ab^	72.75 ± 0.42 ^ab^	12.68 ± 0.32 ^ab^	22.67 ± 0.47 ^ab^	4.26 ± 0.21 ^a^
L45	60.13 ± 0.13 ^ab^	65.63 ± 0.13 ^ab^	72.53 ± 0.10 ^ab^	12.40 ± 0.08 ^ab^	22.71 ± 0.96 ^ab^	4.13 ± 0.11 ^a^
L60	60.35 ± 0.13 ^b^	65.78 ± 0.13 ^bc^	72.63 ± 0.29 ^ab^	12.28 ± 0.22 ^b^	23.83 ± 0.95 ^ab^	4.40 ± 0.26 ^ab^
L120	60.30 ±0.30 ^b^	65.93 ± 0.12 ^cd^	72.97 ± 0.12 ^a^	12.67 ± 0.40 ^ab^	24.56 ± 0.49 ^b^	4.37 ± 0.32 ^ab^
H15	61.05 ± 0.13 ^d^	66.05 ± 0.17 ^cd^	72.58 ± 0.26 ^ab^	11.53 ± 0.29 ^d^	23.00 ± 1.91 ^ab^	4.61 ± 0.48 ^ab^
H30	61.53 ± 0.22 ^e^	66.20 ± 0.18 ^de^	72.45 ± 0.33 ^b^	10.93 ± 0.26 ^c^	21.79 ± 0.51 ^ab^	4.66 ± 0.10 ^ab^
H45	61.78 ± 0.05 ^f^	66.35 ± 0.24 ^e^	72.45 ± 0.13 ^b^	10.68 ± 0.13 ^c^	23.51 ± 0.78 ^ab^	5.15 ± 0.40 ^b^
H60	62.13 ± 0.05 ^c^	66.83 ± 0.17 ^f^	72.73 ± 0.25 ^ab^	10.60 ± 0.22 ^c^	21.37 ± 1.37 ^a^	4.56 ± 0.40 ^ab^
H120	62.78 ± 0.19 ^g^	67.28 ± 0.30 ^g^	72.93 ± 0.46 ^a^	10.15 ± 0.42 ^e^	21.52 ± 1.90 ^ab^	4.80 ± 0.55 ^ab^

Values followed by the same letters in the same column are not significantly different at α < 0.05.

**Table 5:** Thermodynamic characteristics of the retrogradation of the native starch and distarch phosphates.

	T_o2_ (°C)	T_p2_ (°C)	T_c2_ (°C)	ΔT_2_ (°C)	ΔH_2_ (J/g d.w.b.)	PHI_2_	ΔH_2_/ΔH_1_
N	45.30 ± 0.67 ^e^	61.31 ± 0.31 ^f^	72.72 ± 0.30 ^b^	27.41 ± 0.48 ^d^	10.13 ± 0.58 ^ab^	0.63 ± 0.03 ^ab^	0.46 ± 0.02 ^b^
L15	38.63 ± 0.38 ^a^	58.17 ± 0.81 ^a^	71.33 ± 1.37 ^ab^	32.70 ± 1.21 ^a^	10.07 ± 0.33 ^ab^	0.52 ± 0.03 ^ac^	0.44 ± 0.03 ^b^
L30	39.95 ± 0.95 ^bc^	57.68 ± 1.00 ^a^	70.43 ± 0.52 ^a^	30.48 ± 1.26 ^b^	9.42 ± 0.43 ^ab^	0.54 ± 0.07 ^ac^	0.42 ± 0.02 ^ab^
L45	42.63 ± 0.32 ^d^	58.70 ± 0.17 ^ab^	70.30 ± 0.35 ^a^	27.67 ± 0.06 ^cd^	8.94 ± 0.87 ^ab^	0.56 ± 0.06 ^abc^	0.39 ± 0.03 ^ab^
L60	44.57 ± 0.42 ^e^	59.17 ± 0.32 ^bc^	70.83 ± 0.06 ^ab^	26.27 ± 0.38 ^de^	9.34 ± 0.92 ^ab^	0.64 ± 0.09 ^ab^	0.40 ± 0.03 ^ab^
L120	44.95 ± 0.37 ^e^	59.43 ± 0.17 ^bcd^	70.33 ± 0.30 ^a^	25.38 ± 0.45 ^ef^	10.50 ± 1.19 ^b^	0.73 ± 0.09 ^b^	0.43 ± 0.05 ^ab^
H15	39.03 ± 1.29 ^ab^	60.07 ± 0.99 ^cde^	72.00 ± 0.70 ^b^	32.97 ± 0.64 ^a^	10.37 ± 0.70 ^b^	0.49 ± 0.04 ^ac^	0.47 ± 0.04 ^ab^
H30	40.73 ± 0.54 ^c^	60.15 ± 0.35 ^cde^	71.45 ± 0.55 ^ab^	30.73 ± 0.50 ^b^	8.13 ± 0.62 ^a^	0.42 ± 0.03 ^c^	0.37 ± 0.03 ^a^
H45	43.33 ± 0.21 ^d^	60.10 ± 0.66 ^cde^	71.93 ± 1.70 ^b^	28.60 ± 1.50 ^c^	10.27 ± 0.08 ^b^	0.61 ± 0.03 ^ab^	0.44 ± 0.02 ^ab^
H60	44.95 ± 0.90 ^f^	60.18 ± 0.17 ^de^	71.90 ± 0.64 ^b^	26.95 ± 1.35 ^d^	9.21 ± 0.51 ^ab^	0.61 ± 0.05 ^ab^	0.43 ± 0.02 ^ab^
H120	46.50 ± 0.45 ^e^	60.80 ± 0.08 ^e^	71.20 ± 0.37 ^ab^	24.70 ± 0.64 ^f^	7.78 ± 0.68 ^cde^	0.55 ± 0.06 ^d^	0.42 ± 0.02 ^a^

Values followed by the same letters in the same column are not significantly different at α < 0.05.

**Table 6:** Parameter of the Herschel–Bulkley model describing flow curves of native starch and distarch phosphate pastes and recovery.

	Herschel–Bulkley Model				Area of Hysteresis Loop	Recovery (%)
	τ_0_ (Pa)	K (Pa · s^n^)	n	R^2^		
N	5.36 ± 0.41 ^b^	3.08 ± 0.39 ^a^	0.75 ± 0.03 ^a^	0.9991	−169 ± 54 ^a^	140 ± 10 ^c^
L15	2.93 ± 0.39 ^a^	2.98 ± 0.32 ^a^	0.75 ± 0.01 ^a^	0.9992	−167 ± 28 ^a^	114 ± 9 ^b^
L30	2.25 ± 0.21 ^a^	3.90 ± 0.41 ^b^	0.65 ± 0.20 ^b^	0.9988	−44 ± 15 ^b^	133 ± 6 ^c^
L60	2.86 ± 0.16 ^a^	4.69 ±0.10 ^c^	0.63 ± 0.01 ^b^	0.9989	−39 ± 18 ^b^	159 ± 3 ^d^
L120	4.61 ± 0.70 ^b^	3.40 ± 0.10 ^ab^	0.72 ± 0.01 ^a^	0.9979	−160 ± 32 ^a^	133 ± 3 ^c^
H15	2.51 ± 0.50 ^a^	8.51 ± 0.35 ^d^	0.51 ± 0.01 ^c^	0.9994	106 ± 42 ^c^	116 ± 8 ^b^
H30	12.78 ± 0.2 ^c^	9.49 ± 0.98 ^d^	0.60 ± 0.08 ^d^	0.9995	69 ± 53 ^bc^	96 ± 2 ^a^
H60	8.20 ± 1.60 ^d^	31.37 ± 0.15 ^e^	0.48 ± 0.01 ^c^	0.9960	670 ± 128 ^d^	95 ± 2 ^a^
H120	20.88 ± 3.2 ^e^	43.29 ± 2.67 ^f^	0.49 ± 0.01 ^c^	0.9973	1040 ± 57 ^e^	87 ± 2 ^e^

Values followed by the same letters in the same column are not significantly different at α < 0.05.

**Table 7:** Statistically significant Pearson’s correlation coefficients between physicochemical parameters and phosphorus content or modification time.

		Phosphorus Content			Modification Time		
		L-Starch	H-Starch	L + H Starch	L Starch	H Starch	L + H Starch
Casson model *	τ_C0_		0.8688	0.7581		0.9904	0.5143
	η_C_		0.9246	0.6601		0.9681	0.5944
Ostwald de Waele model *	K		0.8742	0.7564		0.9885	0.5183
	n			−0.7867		−0.8902	
Herschel-Bulkley model	τ_0_		0.7529	0.7159	0.8368	0.8212	0.5113
	K		0.8411	0.7347		0.9554	0.4972
	n			−0.6715			
Area of hysteresis loop			0.7985	0.7240		0.9515	
Recovery (R)			−0.9299	−0.5372		−0.7813	
T_o1_		0.5718	0.8688	0.8737	0.5526	0.9514	0.4128
T_p1_		0.5176	0.7310	0.8227	0.7130	0.8950	0.5521
T_c1_						0.4838	0.3875
ΔT_1_			−0.8495	−0.8360		−0.8005	
ΔH_1_					0.4668		
PHI_1_				0.5753			
T_o2_		0.8183	0.8945	0.6796	0.8352	0.8856	0.8556
T_p2_		0.5250		0.7634	0.6380	0.5209	0.4032
T_c2_							
ΔT_2_		−0.8471	−0.8855	−0.5547	−0.8267	−0.9002	−0.8668
ΔH_2_							
PHI_2_		0.7292	0.5869		0.7566	0.6351	0.6690

* The equation parameters are listed in Table S2 in Supplementary Materials.

## Data Availability

The data presented in this study are available on request from the corresponding author.

## Supplementary Materials

The following are available online at https://www.mdpi.com/article/10.3390/polym13152548/s1. Table S1: Statistical analysis of color (SPEX, D65, CIE 1964), Table S2: Rheological analysis of starch pastes (Ostwald de Waele model, Casson model).

## Abbreviations

N	native starch (unmodified)
L	starch obtained in lower temperature of modification (15 °C)
H	starch obtained in higher temperature of modification (45 °C)
15 30 45 60 120	modification time (min)
	Example: L30 means starch phosphate obtained in 30 min of modification at 15 °C.
d.w.b.	dry weight basis
