# Supplementary material for: Effect of Esterification Conditions on the Physicochemical Properties of Phosphorylated Potato Starch
Source: Polymers (Basel). 2021 Jul 31;13(15):2548. doi: 10.3390/polym13152548 (PMC8347770; doi:10.3390/polym13152548)
Supplement: Supplementary file 1 [file polymers-13-02548-s001.zip › polymers-1303647-supplementary.pdf]

polymers

MDPI

Supplementary Materials

Effect of Esterification Temperature and Duration on the Physicochemical Properties of Phosphorylated Potato Starch

Jacek Rożnowski *, Lesław Juszczak, Barbara Szwaja-and Izabela Przetaczek-Rożnowska

Department of Food Analysis and Evaluation of Food Quality, Faculty of Food Technology, University of Agriculture in Krakow, Balicka 122 Str., 30-149 Krakow, Poland; rrjuszcz@cyf-kr.edu.pl (L.J.); barbara.szwa2000@op.pl (B.S.); izabela.przetaczek-roznowska@urk.edu.pl (I.P.-R.)

* Correspondence: rrroznow@cyf-kr.edu.pl; Tel.: +48-12-662-47-46

Received: 1 July 2021; Accepted: 27 July 2021; Published: 31 July 2021

Table S1. Statistical analysis of color (SPEX, D65, CIE 1964).

	L*	a*	b*	C*	h°	WI$_C$	YI	TI	WI$_J$	WI$_U$
N	93.41 ± 0.02^{a}	-0.23 ± 0.02^{a}	2.11 ± 0.05^{ab}	2.12 ± 0.05^{ab}	96.18 ± 0.56^{a}	73.70 ± 0.22^{a}	3.92 ± 0.08^{ab}	-0.42 ± 0.03^{a}	92.98 ± 0.02^{a}	73.35 ± 0.23^{a}
L15	92.70 ± 0.41^{d}	-0.10 ± 0.01^{d}	2.23 ± 0.04^{cde}	2.23 ± 0.04^{cd}	92.55 ± 0.22^{de}	71.98 ± 0.10^{d}	4.27 ± 0.06^{de}	-0.68 ± 0.02^{ef}	92.37 ± 0.04^{c}	71.04 ± 0.14^{de}
L30	92.66 ± 0.09^{d}	-0.14 ± 0.03^{bcd}	2.16 ± 0.01^{ac}	2.16 ± 0.01^{ac}	93.59 ± 0.61^{cde}	72.18 ± 0.17^{cd}	4.11 ± 0.04^{cd}	-0.60 ± 0.05^{cde}	92.35 ± 0.08^{c}	71.47 ± 0.08^{cde}
L45	92.75 ± 0.09^{d}	-0.09 ± 0.01^{d}	2.27 ± 0.02^{de}	2.27 ± 0.03^{de}	92.28 ± 0.36^{e}	71.89 ± 0.12^{d}	4.36 ± 0.04^{e}	-0.72 ± 0.02^{ef}	92.40 ± 0.08^{c}	70.86 ± 0.05^{e}
L60	92.92 ± 0.02^{bcd}	-0.12 ± 0.03^{cd}	2.13 ± 0.01^{ab}	2.13 ± 0.01^{ab}	93.09 ± 0.73^{de}	72.96 ± 0.09^{abc}	4.05 ± 0.04^{bc}	-0.62 ± 0.05^{def}	92.61 ± 0.02^{bc}	72.20 ± 0.21^{bc}
L120	92.87 ± 0.10^{bcd}	-0.12 ± 0.03^{cd}	2.17 ± 0.02^{ac}	2.18 ± 0.01^{ac}	93.16 ± 0.71^{cde}	72.63 ± 0.30^{bcd}	4.14 ± 0.03^{cd}	-0.63 ± 0.04^{def}	92.55 ± 0.10^{bc}	71.84 ± 0.31^{bcd}
H15	93.12 ± 0.19^{bc}	-0.20 ± 0.01^{ab}	2.04 ± 0.01^{a}	2.05 ± 0.01^{b}	95.57 ± 0.27^{ab}	73.80 ± 0.48^{a}	3.82 ± 0.02^{a}	-0.44 ± 0.02^{ab}	92.82 ± 0.18^{b}	73.41 ± 0.49^{a}
H30	92.80 ± 0.05^{cd}	-0.16 ± 0.02^{abcd}	2.12 ± 0.03^{ab}	2.13 ± 0.04^{ab}	94.32 ± 0.39^{abcd}	72.68 ± 0.05^{bcd}	4.02 ± 0.05^{bc}	-0.54 ± 0.02^{bcd}	92.49 ± 0.04^{c}	72.09 ± 0.02^{bc}
H45	92.81 ± 0.02^{cd}	-0.18 ± 0.02^{abc}	2.04 ± 0.01^{a}	2.04 ± 0.02^{b}	95.05 ± 0.39^{abc}	73.11 ± 0.09^{ab}	3.84 ± 0.02^{a}	-0.48 ± 0.03^{abc}	92.53 ± 0.02^{bc}	72.65 ± 0.08^{ab}
H60	92.90 ± 0.08^{bcd}	-0.15 ± 0.02^{bcd}	2.18 ± 0.02^{acd}	2.19 ± 0.01^{acd}	93.92 ± 0.43^{bcde}	72.67 ± 0.18^{bcd}	4.13 ± 0.02^{cd}	-0.58 ± 0.03^{cde}	92.57 ± 0.08^{bc}	71.99 ± 0.22^{bc}
H120	92.96 ± 0.08^{bcd}	-0.15 ± 0.02^{bcd}	2.32 ± 0.02^{e}	2.32 ± 0.01^{e}	93.79 ± 0.45^{bcde}	72.17 ± 0.26^{cd}	4.39 ± 0.02^{e}	-0.63 ± 0.02^{def}	92.59 ± 0.08^{bc}	71.38 ± 0.22^{cde}

Values followed by the same letters in the same column are not significantly different at $\alpha < 0.05$ level.

polymers

MDPI

Table S2. Rheological analysis of starch pastes (Ostwald de Waele model, Casson model).

	**Ostwald – de Waele model (power-law)**			**Casson model**		
	**K**	**n**	**R^2**	**τ_{0C}**	**η_C**	**R^2**
N	6.20 ± 0.25[ab]	0.61 ± 0.01[a]	0.9940	5.40 ± 0.29[ab]	0.57 ± 0.01[a]	0.9922
L15	4.75 ± 0.56[c]	0.66 ± 0.02[c]	0.9982	3.45 ± 0.61[c]	0.60 ± 0.01[a]	0.9924
L30	5.38 ± 0.56[ac]	0.59 ± 0.01[ab]	0.9993	4.68 ± 0.68[ac]	0.43 ± 0.00[b]	0.9734
L60	6.57 ± 0.21[b]	0.57 ± 0.01[b]	0.9991	6.06 ± 0.38[b]	0.45 ± 0.02[b]	0.9737
L120	6.19 ± 0.41[ab]	0.61 ± 0.00[a]	0.9969	5.34 ± 0.38[ab]	0.56 ± 0.04[a]	0.9848
H15	10.32 ± 0.01[d]	0.48 ± 0.00[b]	0.9990	11.21 ± 0.02[d]	0.34 ± 0.01[c]	0.9666
H30	17.90 ± 1.05[e]	0.49 ± 0.00[b]	0.9941	20.02 ± 1.07[e]	0.63 ± 0.06[a]	0.9867
H60	37.46 ± 1.28[f]	0.45 ± 0.00[a]	0.9973	42.73 ± 1.36[f]	1.00 ± 0.07[d]	0.9434
H120	58.39 ± 0.89[g]	0.44 ± 0.01[a]	0.9977	68.51 ± 1.88[g]	1.38 ± 0.06[e]	0.9578

Values followed by the same letters in the same column are not significantly different at $\alpha < 0.05$ level.
